# Influence of battery power setting on carbonyl emissions from electronic cigarettes

**DOI:** 10.18332/tid/126406

**Published:** 2020-09-14

**Authors:** Zuzana Zelinkova, Thomas Wenzl

**Affiliations:** 1 Joint Research Centre, European Commission, Geel, Belgium

**Keywords:** emission, electronic cigarettes, vaping, carbonyls, power setting

## Abstract

**INTRODUCTION:**

Although e-cigarettes share common features such as power units, heating elements and e-liquids, the variability in design and possibility for customization represent potential risks for consumers. A main health concern is the exposure to carbonyl compounds, which are formed from the main components of e-liquids, propylene glycol and glycerol, through thermal decomposition. Levels of carbonyl emissions in e-cigarette aerosols depend, amongst others, on the power supplied to the coil. Thus, e-cigarettes with adjustable power outputs might lead to high exposures to carbonyls if the users increase the power output excessively. The aim of this work was to elucidate the generation of carbonyls in relation to undue battery power setting.

**METHODS:**

Carbonyl emissions were generated by two modular e-cigarettes equipped with two atomizers containing coils of different resistance following the ISO 20768:2018 method. The battery power output was increased from the lower wattage level to above the power range recommended by the producer. Carbonyls were trapped by a 2,4-dinitrophenylhydrazine (DNPH) solution and analysed by LC-MS/MS.

**RESULTS:**

The amount of carbonyl emissions increased with increasing power setting. An exponential incline was observed when the applied power level exceeded the recommended power range. Exceeding the recommended power range by just 5 watts resulted in up to twenty times the amount of carbonyls emitted at the recommended upper power level. Generation of acetaldehyde and acrolein next to other carbonyls was prominent at high power outputs.

**CONCLUSIONS:**

E-cigarettes with customisable power setting might generate high amounts of carbonyls if the battery power output is set by the consumer to levels above the recommended range. This represents a high risk of exposure to carbonyls and thus should be avoided by integrating safety features in e-cigarette devices to limit the possible power settings to the range specified by the manufacturer.

## INTRODUCTION

Electronic cigarettes (e-cigarettes) are characterised by a rapid technological evolution and fast increase in popularity^1^. Products in numerous design variants are currently available on the market^2^. Many e-cigarette devices enable users to modify the character of delivered aerosols by applying atomizers of different resistance and/or adjusting the battery power output^2^.

The main ingredients of e-cigarette liquids are propylene glycol (PG) and glycerol (GLY). Under thermal load, PG and GLY undergo predominantly dehydration and oxidation reactions, which lead to the formation of carbonyls^3^. Carbonyl emissions from e-cigarettes have generated a lot of interest and have been reviewed elsewhere^4,5^. Reported levels of carbonyls varied significantly, mainly due to different vaping parameters applied for aerosol generation^4^. Parameters such as puff volume, puff duration, and puff frequency have an impact on the amount of emitted carbonyls^4,6-8^. To achieve a high level of comparability of results, ISO 20768:2018^9^ defines standard operating conditions for the testing of e-cigarette devices by application of vaping robots.

Increasing the power output of the device results in increased generation of carbonyls^10-12^. Degradation of PG and GLY leads to the generation of a number of compounds such as formaldehyde, acetaldehyde, acetone, dihydroxyacetone, acrolein, acetol, lactaldehyde and other dehydration products^3^. It was also noticed that acrolein in e-cigarette emissions comes mainly from GLY decomposition^13^. The generation of formaldehyde and acetaldehyde from GLY starts at lower temperatures compared to PG^13^. However, the power output provided by latest generation devices allows commercializing e-liquids with a high proportion of GLY in their formulation (even up to 100 %), which might elevate the exposure of consumers to toxic compounds. Formaldehyde is classified by the International Agency for Research on Cancer (IARC) in Group 1 (human carcinogen), acetaldehyde in Group 2B (possible human carcinogen)^14^.

The ability of e-cigarette power units to deliver a large range of power levels to the atomizer does not imply that atomizers can be used at any power setting^4^. Unfortunately, not all producers of atomizers provide clear power specifications. However, the possibility to set the power levels of certain devices above the manufacturer’s specification might lead to an increased risk of carbonyls exposure. Therefore, this study aimed to elaborate the influence of elevated battery power output levels on emitted carbonyls, knowing that the experimental parameters might not always generate aerosols sensorially acceptable for consumers^4^.

## METHODS

E-cigarette VooPoo Drag (device A) was obtained from retail in Belgium, which was supplied with 0.25 Ω NotchCoil^TM^ SS316 (30–70 W) and 0.5 Ω SS316 (15–30 W) Joyetech Cubis Pro coils. The second device Vaporesso SWAG (device B) was purchased via e-commerce in the Netherlands together with two GT Core coils of 0.15 Ω GT4 (30–70 W) and 0.5 Ω GT cCELL (15–40 W) specified by the manufacturer for this device. The two e-cigarettes comprised a refillable reservoir and a battery with customisable power setting. The power supply of device A is able to deliver a nominal power from 5 to 157 W. Device B can deliver a power between 5 and 80 W. A large amount of nicotine-free tobacco flavoured e-liquid (Flavormonks, Belgium) was mixed with nicotine base (nicotine 20 mg/mL, Extra Pure). The mixed e-liquid contained an equal proportion of PG and GLY.

Aerosols were generated on a LM4E linear vaping machine for e-cigarettes (Borgwald KC GmbH, Hamburg, Germany) according to ISO 20768:2018^9^. As specified in the standard, the puffing parameters of 55 mL puff volume, 3 s puff duration and 30 s puff frequency were used. In total 20 puffs were collected for device A. Due to a smaller volume of the reservoir of device B (2 mL), the amount of collected puffs was lowered to 10 puffs.

Determination of carbonyls in emission was carried out based on CORESTA recommended method no. 74^15^. The analytical standards of carbonyl-DNPH derivatives (formaldehyde, acetaldehyde, acrolein, acetone, propionaldehyde, crotonaldehyde, 2-butanone, and butyraldehyde) were purchased from Sigma Aldrich (Overijse, Belgium). All other chemicals and solvents were obtained from Sigma Aldrich and VWR (Leuven, Belgium). Glass impinger flasks filled with glass beads (25 g) and DNPH solution (15 mL) were used to trap the carbonyl emissions. The mouthpiece of the e-cigarette was connected to the impinger with a silicon tube. After aerosol collection, the tube was rinsed with 1 mL of DNPH solution. Carbonyl derivatives were analysed by LC-MS/MS, which consisted of an Agilent 1100 series HPLC system (Agilent Technologies, Santa Clara, USA) equipped with an Acclaim^TM^ Carbonyl C18 column (2.2 μm, 2.1 × 150 mm, Thermo Scientific, Merelbeke, Belgium) and a Micromass Quattro Ultima^TM^ PT mass spectrometer (Waters, Milford, USA) operated by MassLynx V4.1 software (Waters). Details on the analytical method are given in Supplementary file Table S1. The mass of collected aerosol was determined gravimetrically by weighing the cartomizer before and after puffing.

## RESULTS

**Figure 1:**
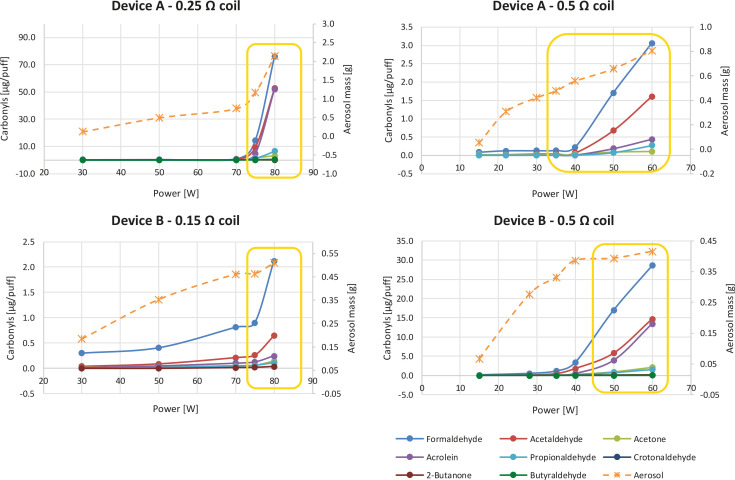
Generation of carbonyl emissions (expressed per puff) by device A and device B equipped with the dedicated coils (heading of each graph) and amount of aerosol collected per one puff session (20 puffs for device A; 10 puffs for device B). Yellow frames indicate the power setting above the recommended power range of the coils

The power of e-cigarettes was increased from the lower level of the power range specified for the particular atomizer to above the specified power range. The amount of carbonyl emissions increased with increasing power, as did the amount of generated aerosol. Figure 1 displays the development of carbonyls and the emitted aerosol mass. The amounts of carbonyls emitted by the two devices at power levels above specifications increased markedly. Device A equipped with a 0.25 Ω coil was characterised by an increase of carbonyl emissions from 0.3 to 190.9 μg/puff (sum of 8 carbonyls) when the power was increased from 30 to 80 W. Interestingly, the increase of just 5 watts above the specification range resulted in about a 20 times higher amount of carbonyl emissions. Lower carbonyl levels (from 0.1 to 5.5 μg/puff) were observed for device A in combination with a 0.5 Ω coil at power settings from 15 to 60 W. Controversial results were obtained for device B. Equipped with a low resistance coil (0.15 Ω), it emitted lower levels of carbonyls (0.4 to 3.3 μg/puff; power range 30–80 W), compared to the combination with a 0.5 Ω coil (0.1 to 60.7 μg/puff; power setting 15–60 W).

The composition of generated carbonyls was not constant over different power levels. In general, formaldehyde represented the major component of carbonyl emissions (between 60% to 100% at the lowest power settings). However, the contribution of formaldehyde decreased with increasing wattage (between 33% and 64% at the highest power levels). This is caused by the additional formation of other carbonyl compounds, which were not detected at the low power setting or were present only in traces. The most obvious increase in terms of relative composition was observed for acetaldehyde (from not detected to 31%) and acrolein (from not detected to 30%). The composition of carbonyls detected at different power settings is given in Supplementary file Figure S1.

## DISCUSSION

It is well known that the power setting of the e-cigarettes has an impact on the formation of carbonyl compounds. A number of studies showed an increase of carbonyl emissions at the upper power levels specified for the particular coil employed^10,12^. This raised the question of what happens above the specified power range. Although the e-cigarette should never be operated outside the recommended power setting, the modularity of several devices opens the possibility to exceed, wittingly or unwittingly, the applicable power range. In this study, we did not make any assumptions on the consumer acceptance as the conducted tests focused solely on the carbonyl emission levels.

The obtained results confirmed the influence of power supplied to the atomizer on carbonyl emissions. The levels of carbonyls increased slightly with increasing power levels within the recommended power range. However, an exponential increase was observed when the power level exceeded the recommended power range. The increase in carbonyl emissions can be partially explained by the increasing amount of aerosolized e-liquid per puff. However, the amounts of carbonyls continue to increase exponentially with power, even when corrected by the mass of the aerosolised e-liquid.

The results are consistent with the Wang et al.^13^ findings. A significant increase was observed for formaldehyde, acetaldehyde and acrolein with temperature increase. The authors demonstrated that the evolution of formaldehyde and acetaldehyde from PG/GLY mixture was initiated at a relatively low temperature (108°C and 215°C, respectively). However, the development of acrolein began at higher temperature (270°C)^13^. Higher battery output, and thus higher coil temperature, enhanced the degradation of PG and GLY, which resulted in formation of additional carbonyl compounds.

## CONCLUSIONS

The study demonstrated that the power setting of the e-cigarette has a significant impact on carbonyl emissions, which might increase exponentially when the device is operated above the power range specified by the manufacturer. Both tested devices are compatible with several atomizers with different recommended power setting. It is not unlikely that users confuse power settings for different atomizers or change them accidentally. Therefore, the integration of safety features should be considered for e-cigarette devices with customisable power settings in order to prevent exceedance of the recommended power range of the installed atomizer.

## CONFLICTS OF INTEREST

The authors have completed and submitted the ICMJE Form for Disclosure of Potential Conflicts of Interest and none was reported.
