# Supplementary material for: Influence of battery power setting on carbonyl emissions from electronic cigarettes
Source: Tob Induc Dis. 2020 Sep 14;18:77. doi: 10.18332/tid/126406 (PMC7528267; doi:10.18332/tid/126406)
Supplement: Supplementary file 1 [file TID-18-77-s1.pdf]

Supplementary Table S1. Parameters of the LC-MS/MS method for carbonyl determination.

Mobile phase	A: water<br>B: acetonitrile		
Flow	0.3 mL/min		
Gradient	0 min 55% B, 15% B; 10 min 55% B; 15 min 100% B; 18 min 100% B; 18.5 min 55% B; 25 min 55% B		
Injection	5 µL		
MS/MS	Negative ionisation mode<br>Cone: 75 V<br>Capillary: 3.5 kV<br>Desolvation temperature: 320 °C<br>MS/MS operated in selective reaction monitoring mode (SRM)		
SRM parameters	*Transition Q1 (m/z)*[1]	*Transition Q2 (m/z)*	*Collision energy (V)*
Formaldehyde-DNPH	209>151	209>163	5
Acetaldehyde-DNPH	223>151	223>163	7
Acrolein-DNPH	235>158	235>163	9
Acetone-DNPH	237>207	237>151	8
Propionaldehyde-DNPH	237>163	237>152	9
Crotonaldehyde-DNPH	249>172	249>163	10
2-Butanone-DNPH	251>152	251>221	10
Butyraldehyde-DNPH	251>221	251>163	8
Acetaldehyde-D4-DNPH	227>151	227>163	7
Acetone-D6-DNPH	243>213	243>151	8
Cyclopentanone-D4-DNPH	267>237	267>219	12

[1] Transition Q1 was used for the quantification of the compounds.

Supplementary Figure S1. Relative composition of carbonyl emissions generated by A) device A with 0.25 Ω coil (recommended 30-70 W), B) device A with 0.5 Ω coil (recommended 15-30 W), C) device B with 0.15 Ω coil (recommended 30-70 W) and D) device B with 0.5 Ω coil (recommended 15-40 W).

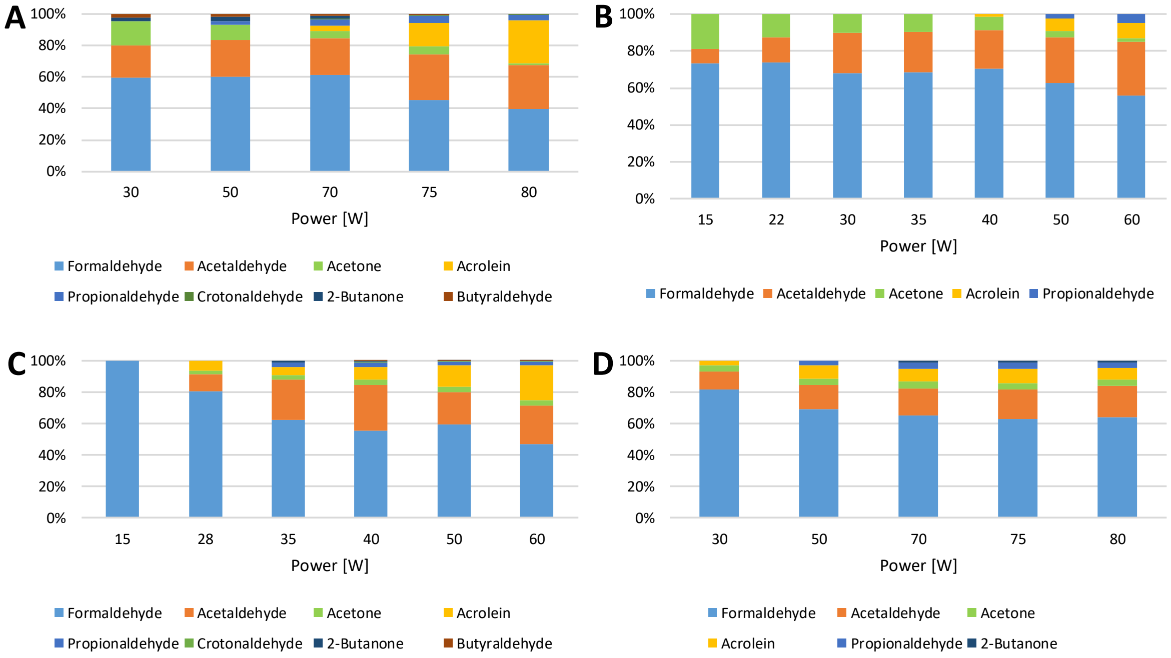

© 2020 Zelinkova Z. and Wenzl T.
